# Case Report: *Kingella kingae* causing prosthetic joint infection in an adult

**DOI:** 10.1099/acmi.0.000559.v3

**Published:** 2023-08-09

**Authors:** Katherine Wensley, Damian McClelland, Natalie Grocott, Gopikanthan Manoharan, Seema Desai

**Affiliations:** ^1^​ Department of Trauma and Orthopaedics, Royal Stoke University Hospital, Stoke-on-Trent ST4 6QG, UK; ^2^​ Department of Microbiology, Royal Stoke University Hospital, Stoke-on-Trent ST4 6QG, UK

**Keywords:** *Kingella kingae*, prosthetic joint infection, immunocompromised

## Abstract

**Introduction.:**

*
Kingella kingae* is a Gram-negative micro-organism that is rarely isolated as a pathogen in the adult population. Although widely reported to affect prosthetic heart valves, there have been no previously reported cases of *K. kingae* infecting prosthetic joints in adults.

**Case Presentation.:**

A 61-year-old patient with a history of rheumatoid arthritis presented with insidious onset of pain and swelling in her right shoulder, which had progressed to a discharging sinus. The patient had undergone a total shoulder replacement 11 years previously and had not developed any prior post-operative infections. She had been taking anti-TNF medication for 5 years prior to review for her rheumatoid disease. The patient underwent a two-stage revision replacement procedure, including implant removal, sinus excision and debridement. Deep tissue samples grew *K. kingae* post-operatively. The patient was commenced on intravenous ceftriaxone for 14 days, followed by a further 28 days of oral ciprofloxacin. A second-stage custom shoulder replacement was undertaken 10 months following the first stage and the patient made a good functional recovery.

**Conclusion.:**

The authors suggest that clinicians should be attuned to *K. kingae* as a potential pathogen for prosthetic joint infection, particularly in patients who are immunosuppressed. Two-stage revision procedures can ensure a favourable outcome and eradication of this pathogen from the joint. Beta lactams remain the principal antibiotic of choice.

## Data Summary

No data were generated, or reused, in this case report.

## Introduction

*Kingella kingae* was previously considered to be part of the normal oropharyngeal flora [1], but due to improved culture techniques for this fastidious organism, it has recently been acknowledged as an emergent pathogen in the paediatric population, where it typically affects bones and joints [2]. There are very few reported cases in the adult population, where it typically causes endocarditis, spondylodiscitis or bacteraemia [3]. We present a previously unreported case of *K. kingae* prosthetic joint infection in a patient with inflammatory joint disease. Clinicians should be attuned to *K. kingae* as a potential cause of prosthetic joint infection, particularly in patients who are immunosuppressed due to immune suppressive medications. Operating surgeons must obtain deep tissue samples using sterile instruments during surgery and send them to a microbiology laboratory for culture and identification of the pathogen.

## Case presentation

**Fig. 1.**
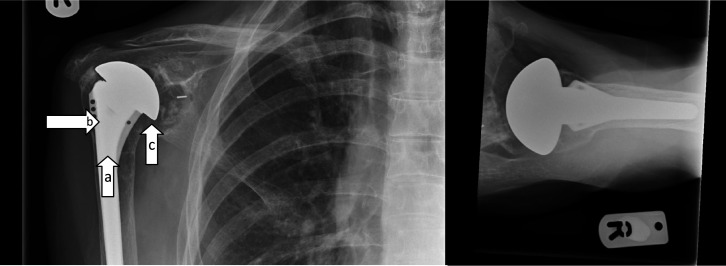
X-rays taken at initial consultation demonstrating elevation of the humeral head (**a**), medialization of the glenoid implant (**b**) and osteolysis around the humeral implant (**c**).

A 61-year-old lady with a history of rheumatoid arthritis since 1986 was referred to the orthopaedic surgical clinic by her treating rheumatologist. She had a total shoulder replacement implanted 11 years previously at a different hospital trust and did not have any delayed healing or episodes of infection post-operatively. On attendance at the orthopaedic clinic, she reported a 1 month history of pain and swelling in the shoulder, which had progressed to an intermittently discharging sinus over the posterior aspect of her shoulder. She had not recently undergone any dental intervention or other surgical procedures, and had otherwise been systemically well throughout this period. X-rays revealed elevation of the humeral head consistent with rotator cuff failure. The replacement head had also migrated medially due to failure of the supporting glenoid bone and there was osteolysis around the humeral implant (Fig. 1.).

The patient had been on immunosuppressive medications, which included anti-TNF medication and methotrexate for rheumatoid arthritis for the previous 5 years, and was not taking antibiotics at the time of presentation to the clinic. Initial superficial wound swabs were taken for culture at this clinic attendance, all of which revealed no growth at this stage.

Initially, the patient did not wish to undergo revision surgery and instead wished to consider management with suppressant antibiotic therapy, but after further consideration, she decided to proceed with a revision procedure. A two-stage procedure was planned that initially comprised implant removal, debridement, temporary spacer insertion and empirical intravenous antibiotic therapy post-operatively, followed by reimplantation of a custom-made prosthesis at a later second stage. At the first-stage operation, the implant was removed, the sinus was excised, and an extensive debridement and washout was performed. A large loculated collection and copious infected tissue were noted. Seven deep tissue samples were obtained with sterile instruments and sent to the microbiology laboratory for culture and sensitivity testing in a sterile container. An antibiotic-loaded cement spacer (model H48G, Synicem Shoulder Spacer, Lima Orthopaedics UK Ltd, Letchworth Garden City, UK) loaded with 1.50 g of gentamicin was inserted to maintain tissue tension and joint alignment. The patient was started empirically on IV flucloxacillin 2 g QDS following surgery since Gram-positive organisms are the commonest pathogens in prosthetic joint infections, while awaiting results from the intra-operative samples.

Tissue samples were homogenized using a vortex machine with the addition of saline broth and were plated on the blood, cystine–lactose–electrolyte-deficient (CLED) and chocolate agar. Further, fastidious anaerobe broth (FAB) was also inoculated for each sample and was read each day for a total of 5 days of incubation. *K. kingae* was isolated from two out of seven tissue samples at 48 h from the blood plate and subsequently identified using matrix-assisted laser desorption/ionization time-of-flight mass spectrometry (MALDI-TOF MS) technology. One out of the two positive tissue samples, with further FAB, isolated a similar pathogen. Ciprofloxacin, amoxicillin and ceftriaxone etests were set up, and the organism was detected to be sensitive to all as per European Committee on Antimicrobial Susceptibility Testing (EUCAST) guidelines. Antibiotics at this stage were rationalized as per microbiology advice and the patient was commenced on intravenous ceftriaxone 2 g once a day for 14 days, followed by a further 28 days of oral ciprofloxacin 500 mg BD (equivalent to twice a day) to complete a total 6-week course. A transthoracic echocardiogram was performed in view of the risk of endocarditis, and this was negative.

The second-stage procedure for implantation of the definitive prosthesis (custom-made Lima shoulder, Lima Orthopaedics UK Ltd, Letchworth Garden City, UK) was performed 10 months following the first stage (with two delays due to anaesthetic concerns and the presence of pressure ulcers). At the second-stage procedure, there was no evidence of an active infective process and implantation of the new custom-made prosthesis was undertaken. Three deep tissue samples were obtained using sterile instruments and sent to the microbiology laboratory for culture and sensitivity testing. Samples were processed in a similar manner as previously stated with additional broth incubation for 5 days. The patient was commenced empirically on intravenous vancomycin, dosed as per the local vancomycin calculator, and co-amoxiclav 1.2 g TDS (three times a day), as per the local hospital guidelines for revision surgery post-operatively. No pathogens were isolated from any deep tissue specimens following a final read at 5 days. The patient was subsequently discussed in the Bone Infection MDT (Multi-Disciplinary Team) meeting and it was advised that the antibiotics be rationalized to oral ciprofloxacin 500 mg BD to complete the 6-week post-operative period because the patient was immune suppressed and had a high risk of relapse.

**Fig. 2.**
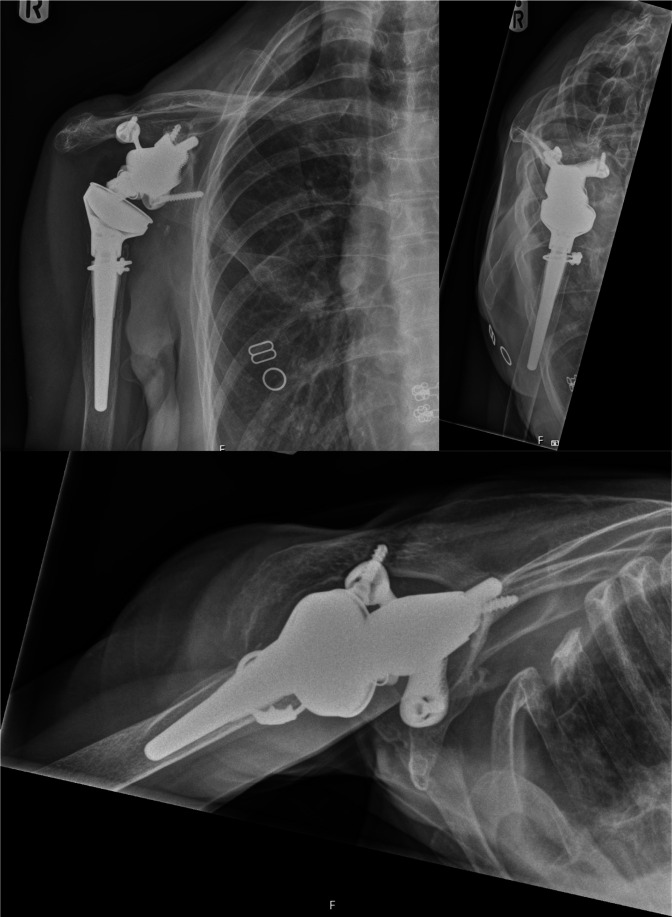
X-ray images taken 12 months post-operatively, demonstrating satisfactory prosthesis position.

The patient completed the course of antibiotics and recovered post-operatively with no further complications from the shoulder perspective. She continued to make progress, and her range of motion at the shoulder joint improved slowly. However, her post-operative physiotherapy was limited to over-the-phone sessions during the coronavirus disease 2019 (COVID-19) crisis and 7 months post-surgery the patient fell, sustaining a closed, right tibial shaft fracture that was successfully managed non-operatively in serial casts, although this further limited her shoulder rehabilitation. Twelve months post-operatively, her radiographs were satisfactory (Fig. 2) and she had regained function to a level beyond her pre-operative range. Her inflammatory markers were CRP 36.6, ESR 59 (consistent with her rheumatoid clinical picture), and WCC 6.8; and her clinical shoulder scores were 36 for the Oxford shoulder score, with a Constant score of 43.

At 14 months post-second stage surgery, the patient was admitted with sepsis, with the likely focus originating from a right foot ulcer that had led to osteomyelitis. Superficial ulcer swabs from the right foot isolated *Staphylococcus aureus* (MSSA). The patient declined any further ongoing treatment and elected to return home with her family for palliative care.

## Discussion

*K. kingae* is a Gram-negative member of the notoriously difficult-to-culture HACEK group (* Haemophilus* species, *Aggregatibacter* species, *Cardiobacterium hominis *, *Eikenella corrodens* and *Kingella* species) of micro-organisms and a component of the normal oropharyngeal flora in children. In the past two decades, we have seen improved laboratory methods for isolating fastidious organisms, leading to increased isolation of rare pathogens from tissue samples. Consequently, there has been greater recognition of its importance as a paediatric pathogen, where it is speculated that *K. kingae* passes into the bloodstream through mucosal lesions caused by upper respiratory tract viral infections. It is known that *K. kingae *, particularly as a fastidious organism, tends to grow slowly on conventional solid culture medium, which might be why the potential of this pathogen in various clinical conditions is underestimated, especially in patients who are immunosuppressed [4]. Additionally, Gram staining on tissues could fail to detect the presence of this organism in a high percentage of cases [2, 5].

More recently, *K. kingae* has been noted as a pathogen in the adult population, most often in immunocompromised individuals, where it has been noted to cause endocarditis, spondylodiscitis or bacteraemia [2]. Although widely reported to affect prosthetic heart valves [6], to our knowledge, there have been no previously reported cases of *K. kingae* infecting prosthetic joints.

The rate of prosthetic shoulder joint infections in the general adult population ranges between 1–4 % [7] and the causative pathogens are usually *Cutibacterium acnes *, *Staphylococcus aureus* and *Staphylococcus epidermidis* [1]. Due to an ageing population and the associated rise in the number of prosthetic shoulder replacements being performed [8], the rate of prosthetic joint infection cases is also likely to increase, and hence knowledge concerning potential pathogens is of increasing consequence.

Collaboration between the surgical team and microbiology colleagues is vital in ensuring optimal management when *K. kingae* is isolated. Penicillins are usually effective in treatment, as are broad-spectrum second- or third-generation cephalosporins [9–11]. Consequently, *K. kingae* is generally susceptible to broad-spectrum empirical antibiotic therapy, which is frequently administered prior to diagnosis due to the slow growth of the organism, delaying pathogen identification. 16S rDNA gene sequencing could assist in identification even after antibiotic use and can significantly reduce time to diagnosis [12]. Alternatively, the literature suggests that identification of *K. kingae* can be further improved by using blood culture broth, whereby joint fluid or pus obtained from the joint is directly inoculated into blood culture broth in theatre, which decreases the concentration of *K. kingae* growth-inhibiting factors normally present in synovial fluid [5]. This technique should therefore be considered in patients with prosthetic joint infection who are deemed to be at higher risk due to being on immunosuppressants.

## Conclusion

*K. kingae* is a rare cause of prosthetic joint infection, but should be considered as a potential pathogen in adults who are immunocompromised. The authors present a case where *K. kingae* was identified as a likely pathogen infecting a primary shoulder joint replacement that was successfully treated with antibiotics and a two-stage revision surgical technique. Multidisciplinary collaboration is vital in optimizing medical and surgical outcomes in such cases.
